# Histopathology of the Intervertebral Disc of *Nothobranchius furzeri*, a Fish Model of Accelerated Aging

**DOI:** 10.3390/biology12101305

**Published:** 2023-10-03

**Authors:** Maria Butylina, Katharina Wahl-Figlash, Michael Kothmayer, Katharina Gelles, Oliver Pusch, Peter Pietschmann

**Affiliations:** 1 Institute for Pathophysiology and Allergy Research (IPA), Center for Pathophysiology, Infectiology and Immunology, Medical University of Vienna, 1090 Vienna, Austria; 2 Center of Anatomy and Cell Biology, Medical University of Vienna, 1090 Vienna, Austria

**Keywords:** *Nothobranchius furzeri*, spinal deformity, intervertebral discs, histology

## Abstract

**Simple Summary:**

Osteoarthritis is a common complex disease, which affects the whole joint and causes disability in millions of patients. Currently, there is no cure for this disease; there is only the possibility of symptomatic therapy. Due to this, animal models play an important role in further investigating the pathophysiology and developing therapeutic strategies. The turquoise killifish, *Nothobranchius furzeri*, is a well-known model for the investigation of the effects of fast aging, that spontaneously develops spinal deformities. The aim of this study was to investigate the characteristics of the intervertebral discs of healthy and deformed *N. furzeri*, which have never been described before. Our study shows age- and disease-related alterations in the vertebral discs of *N. furzeri*.

**Abstract:**

Introduction: Osteoarthritis is a classical age-related disease, which affects millions of patients worldwide. To further understand the pathophysiology and to develop therapeutic strategies for this disease, animal models play a significant role. *Nothobranchius furzeri* is an established model for accelerated aging that spontaneously develops spinal deformities. Although the bone properties of *N. furzeri* are well described, characteristics of the intervertebral discs are still unknown. The aim of this study was to investigate the characteristics of the intervertebral discs of healthy and deformed *N. furzeri*. Material and Methods: Intervertebral properties of healthy and deformed *N. furzeri* were investigated in 8-, 12-, 18- and 21.5-week-old male fish of the GRZ strain. For histological evaluations the fish were decalcified, paraffin-embedded and stained with (1) hematoxylin and eosin, (2) toluidine blue and (3) alcian blue/picrosirius red. Results: 8-week-old and deformed *N. furzeri* showed spongy-like tissue containing vacuolated notochord cells and a beginning formation of fibrous tissue in the central area. Older healthy fish showed fibrous tissue in the central region and a spongy-like tissue in the peripheral region. Conclusion: Our study revealed age- and disease-related alterations of the vertebral discs in *N. furzeri*. Further studies should investigate the utility of *N. furzeri* as a model for degenerative spine diseases.

## 1. Introduction

Osteoarthritis (OA) is known as the most common form of arthritis, which affects more than 500 million people worldwide [1]. It is described as a complex disease, affecting the whole synovial joint, and causes disability in patients due to pain. In general, two different types of OA can be distinguished, primary (idiopathic) and secondary OA. Primary OA occurs naturally due to degenerative changes present in the joints. It can be further differentiated into localized and generalized OA. Localized OA affects only one joint, and the generalized type affects three or more joints. Secondary OA arises due to an underlying condition like trauma, congenital diseases or other diseases and disorders associated with metabolism or the bone, leading to OA in the joint [2,3]. Molecular and metabolic derangements are described as potential reasons for anatomical and/or physiological changes in the affected joints. Such changes include, for example, joint space narrowing, subchondral sclerosis, subchondral cysts, degeneration of cartilage, abnormal bone remodeling, joint inflammation or osteophyte formation [3,4]. Abnormalities in the function of the four components of the synovial joint (articular cartilage, meniscus, subchondral bone and synovial membrane) are involved in promoting OA in the joints [5]. Furthermore, inflammatory mechanisms are present due to an injury caused by mechanical stimulation of the joint. This leads to a release of cytokines, degradative enzymes and collagenases/matrix metalloproteinases (MMPs) by chondrocytes, osteoblasts and synoviocytes. MMPs lead to degradation of the articular cartilage, as they are responsible for collagen matrix degradation. Apart from this, chondrocytes undergo hypertrophy and the subchondral bone undergoes abnormal remodeling, which leads to the formation of subchondral cysts and osteophytes [6,7,8]. Moreover, the innate immune system plays an important role in OA, by activation of the complement and alternative pathways [9].

Degenerative processes of the spine not only include OA of the facet joints but also alterations of the intervertebral discs [10,11,12]. In the early phases of disc degeneration a balance shift of anabolic and catabolic activity of extracellular matrix molecules and chondrocytes occurs, which also involves the presence of pro-inflammatory cytokines, including IL-1, IL-2, IL-4, IL-6, IL-8, IL-10, IL-12, IL-15, IL-17, interferon-γ and TNF-α [13,14]. Pravdyuk et al. described the presence of IL-1β, IL-6 and IL-17 in all discs of degenerative disc disease patients, which was associated with the histological stage of the intervertebral disc degradation [14].

A common symptom of spine diseases is back pain, which frequently is caused by degenerative lesions of the spine [14,15]. Lumbar spondylolisthesis, defined as a slip of a lumbar vertebral body relative to the vertebral body below, may also be involved in the degeneration of the spinal structure [16].

Currently, there is no cure for this disease, but rather a treatment of the symptoms, like pain or immobility, with pain medication and lifestyle amendments. A further treatment option in severe OA cases is a partial or total joint replacement. During the previous years, a high number of treatment modalities has been investigated for the management of low back pain, including an approach using platelet-rich plasma (PRP) [12]. To further understand the pathophysiology and therapeutic efficacy of the treatment for this disease, animal models are inevitable [3]. 

For OA, traditionally small and large animal models have been investigated. Typical small animal models are rodents like mice, rats, rabbits and guinea pigs, which are primarily used to study the pathogenesis and pathophysiology of OA. Large animal models include dogs, goats, sheep and horse, which have a similar anatomy to humans and are used to study the disease process and efficacy of treatment options [3]. As well as rodent and large animal models, fish models like the zebrafish (*Danio rerio*) have been described. Small teleost fish like the *Danio rerio* are vertebrates, which show strong similarities in their skeletal physiology to mammals, including all the main cell types like osteoblasts, osteocytes and chondrocytes, which are present in bone and cartilage tissue [17]. An advantage of using the zebrafish as a model for OA is the possibility of investigating dynamic OA-related processes in vivo, including the differentiation of cartilage in larvae or adult zebrafish. Furthermore, zebrafish express, in their early developmental stages, several genes associated with OA susceptibility in humans, leading to a link between OA and early gene dysfunction during cartilage maturation and maintenance [18,19].

Another small teleost fish, which is an established model for accelerated aging, is the turquoise killifish *Nothobranchius furzeri (N.furzeri)*, which belongs to the group of *Nothobranchius* species [20]. It inhabits seasonal freshwater pools in the southeast of Africa and is known as “the shortest-lived vertebrate that can be kept in captivity” due to their very short life cycle. Furthermore, previous publications in many aspects described similarities of aging between humans and *N.furzeri*, including telomere shortening, decreased mitochondrial numbers, lipofuscin accumulation, hypertrophy of cardiomyocytes, defective vision, abnormal spine curvature and decreased locomotor activity [21,22]. In a previous publication we characterized *N. furzeri* as a model of age-related musculoskeletal diseases. During our previous investigations we detected osteoblasts and mono- and multinucleated osteoclasts, but no osteocytes, in the vertebral area. Furthermore, relative values of the bone volume density decreased with age in male killifish. In our previous study we were able to investigate not only healthy *N. furzeri* but also killifish with visible vertebral deformities, which showed a decrease in the bone volume density, compared to the healthy group [23]. Although we were able to investigate the bone properties of *N. furzeri*, the histology and characteristics of the intervertebral discs and tissues are still unknown. The aim of this study was to investigate and compare the composition and characteristics of the intervertebral discs and tissues of healthy and deformed *N. furzeri*.

## 2. Material and Methods

### 2.1. Animals 

In this study, male *N. furzeri* of the Gone Re Zhou strain were used. Fish were raised at the *Nothobranchius* fish facility, Center of Anatomy and Cell Biology, Medical University of Vienna. Maintenance, mating and breeding were performed as previously described [24]. Briefly, fish were grown in an overflow custom-made system maintained at 27 °C on a 12 h light/dark cycle. As male fish show aggressive behavior towards each other, they were kept in separate tanks. The fish were fed twice daily with commercially available frozen blood worms (*Chironomus larvae)* and monitored for well-being and spontaneous locomotor activity. 

Selected fish were euthanized at 8, 12, 18 and 21.5 weeks of age using tricaine methanesulfonate (MS-222) buffered with sodium bicarbonate to a neutral pH, fixed for 48 h at room temperature with 4% paraformaldehyde, and afterwards stored in 70% ethanol at 4 °C. All killifish were kept and handled according to institutional and national guidelines and laws (2022-0.238.324).

### 2.2. Paraffin Embedding

For paraffin embedding, we decalcified the *N. furzeri* with trisaminomethane–ethylenediaminetetraacetic acid for two days at room temperature (RT) and afterwards dehydrated with increasing alcohol concentrations (80%, 96% and 100%) at RT for 1 h each. Before paraffin embedding, samples were stored in xylol for 1 h at RT. Afterwards, the samples were infiltrated with paraffin and embedded as described previously [25]. Sectioning was performed with the Microtome Microm HM355S (Thermo Scientific, Waltham, MA, USA), using specific knives for hard materials like bones. 

### 2.3. Hematoxylin and Eosin Staining

To obtain the vertebral characteristics, formaldehyde-fixated and paraffin-embedded *N. furzeri* were stained with hematoxylin and eosin (HE) (ROTH, Karlsruhe, Germany), according to the manufacturer’s protocol. 

### 2.4. Toluidine Blue Staining 

Selected paraffin sections of *N. furzeri* were stained with toluidine blue (Fluka Analytical, Sigma-Aldrich, Taufkirchen, Germany) to visualize osteoblasts in the vertebral area. One percentage toluidine blue solution (pH = 4.5) was 1:50 diluted and applied for 2 min.

### 2.5. Alcian Blue/Picrosirius Red Staining

We performed alcian blue/picrosirius red staining (Morphisto, Offenbach am Main, Germany) on paraffin sections to visualize the glycosaminoglycans and collagen type I and III in the vertebral area of the *N. furzeri*. This staining was performed according to the manufacturer’s protocol. 

## 3. Results

**Figure 1:**
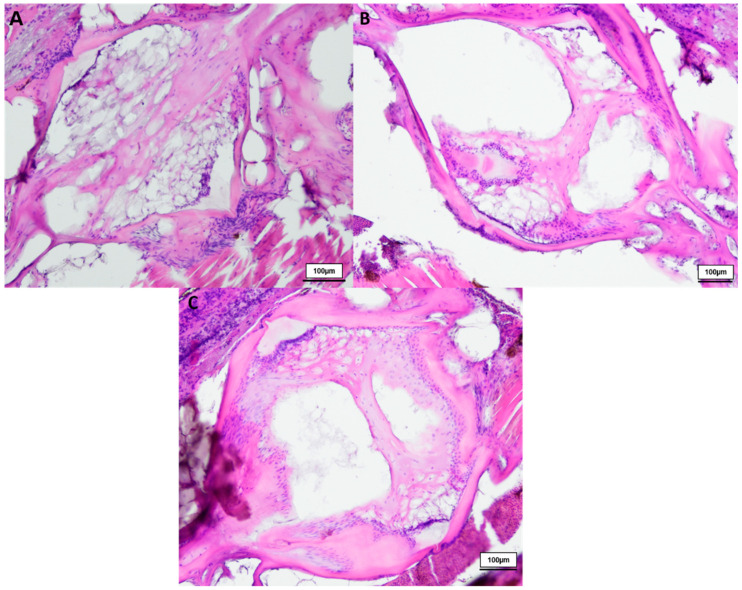
HE-stained vertebral body with intervertebral discs of healthy (**A**) 8-week-old (*n* = 3), (**B**) 12-week-old (*n* = 3) and (**C**) 18-week-old (*n* = 3) male *N. furzeri*, original magnification 100×. (Scale bar = 100 µm).

**Figure 2:**
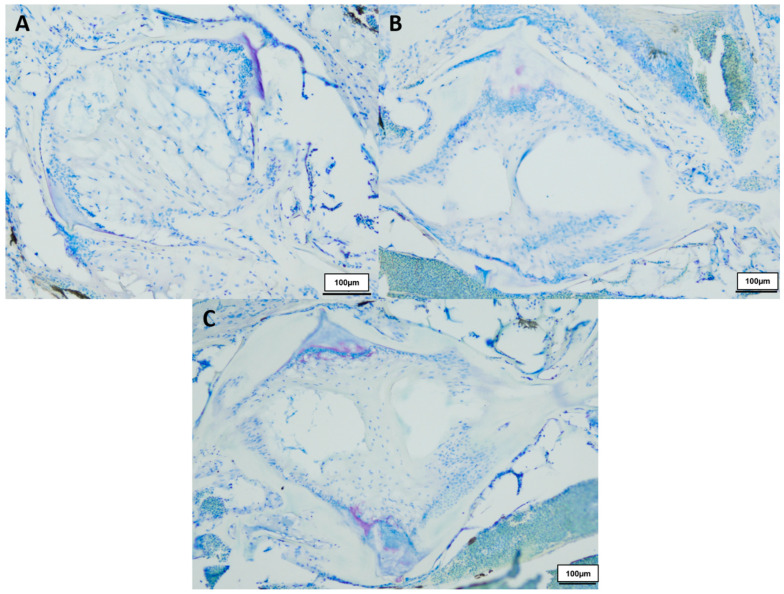
Toluidine blue-stained vertebral body with intervertebral discs of healthy (**A**) 8-week-old (*n* = 3), (**B**) 12-week-old (*n* = 3) and (**C**) 18-week-old (*n* = 3) male *N. furzeri*, original magnification 100×. (Scale bar = 100 µm).

In total we investigated three 8-week-, three 12-week- and three 18-week-old male killifish. Additionally, four 21.5-week-old male killifish, with visible vertebral deformations, were examined. Vertebral deformations occur spontaneously in approximately 1–2% of the background population. During our experiment, we observed reduced locomotor activity in the older fish population, which occurs mostly at around 20 weeks of age. Figure 1 shows the differences in the intervertebral area between healthy young and old *N. furzeri.* Comparing them with each other, younger fish (8 weeks) showed mostly a spongy-like tissue containing vacuolated notochord cells and a beginning formation of fibrous tissue inside the vertebrae. Older fish (12 weeks and 18 weeks) showed fibrous tissue in the central region and a spongy-like tissue in the peripheral region, which contains regressing vacuolated notochord cells. Next to the intervertebral ligament, osteoblasts were visible, which are present in higher numbers in younger than older killifish, as similarly observed in our previous publication (for review see [23]). Similar results were visible in the toluidine blue staining (see Figure 2).

**Figure 3:**
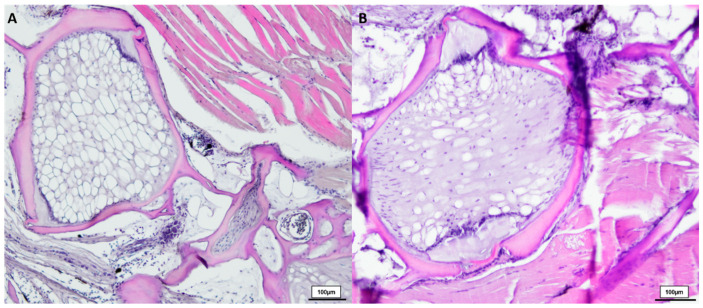
HE-stained vertebral body with intervertebral discs of two deformed 21.5-week-old (*n* = 4) male *N. furzeri* (**A**,**B**), original magnification 100×. (Scale bar = 100 µm).

**Figure 4:**
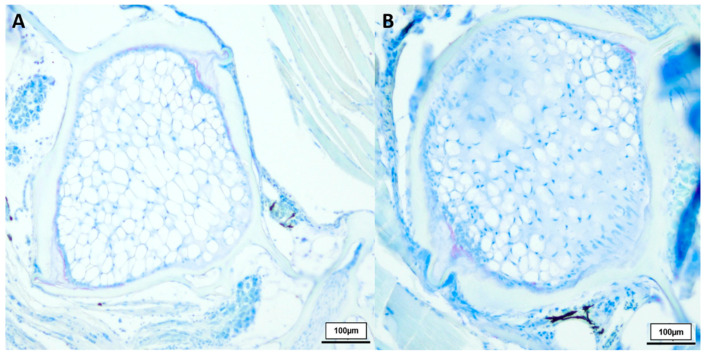
Toluidine blue-stained vertebral body with intervertebral discs of two deformed 21.5-week-old (*n* = 4) male *N. furzeri* (**A**,**B**), original magnification 100×. (Scale bar = 100 µm).

Comparing healthy male *N. furzeri* with the deformed fish (shown in Figure 3), we observed similarities between the 8-week-old and deformed killifish. *N. furzeri* with vertebral deformations also showed a spongy-like structure, containing vacuolated notochord cells, but here no formation of fibrous tissue in the intervertebral area was detected. Toluidine blue-stained sections revealed similar results, shown in Figure 4.

**Figure 5:**
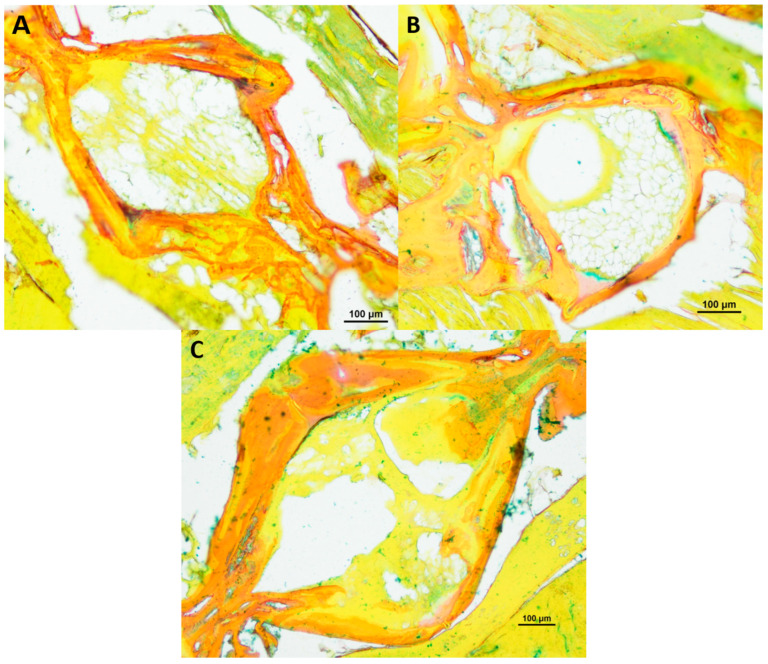
Alcian blue/picrosirius red-stained vertebral body with intervertebral discs of healthy (**A**) 8-week-old (*n* = 3), (**B**) 12-week-old (*n* = 3) and (**C**) 18-week-old (*n* = 3) male *N. furzeri*, original magnification 100×. (Scale bar = 100 µm).

**Figure 6:**
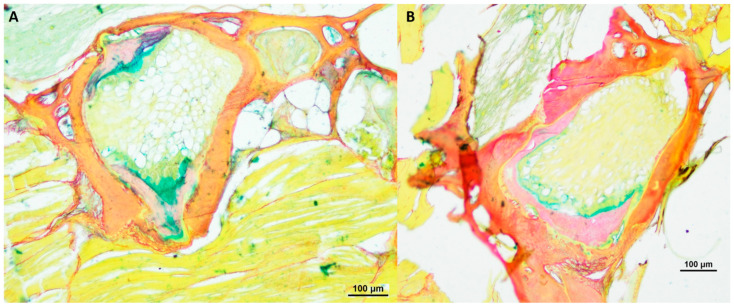
Alcian blue/picrosirius red-stained vertebral body with intervertebral discs of two deformed 21.5-weeks-old (*n* = 4) male *N. furzeri* (**A**,**B**), original magnification 100×. (Scale bar = 100 µm).

Furthermore, we conducted a comprehensive analysis of the intervertebral area in *N. furzeri* using alcian blue/picrosirius red staining, as illustrated in Figure 5 and Figure 6. This specific staining, for the visualization of glycosaminoglycans and collagen type I and III, revealed that the spongy-like structure, containing vacuolated notochord cells, and the fibrous area in the central region seem to consist mostly of type III collagen, as analyzed additionally with polarizing optics (results not shown). These structures were found especially in older healthy killifish. Furthermore, we detected type III collagen within the trabecular structures of the vertebrae. The bone matrix of *N. furzeri* consisted mainly of type I collagen. We could not detect any differences in regard to age or deformity.

## 4. Discussion

With the aim to investigate and compare the composition and characteristics of the intervertebral discs and tissue of healthy and deformed *N. furzeri*, we performed different histological stainings. During our previous studies, we observed a higher incidence of deformities in male fish compared to females. Additionally, male specimens tend to be larger, making deformities easier to investigate. For these reasons, we chose to focus our investigation on the male population. We detected differences between young and old killifish, and similarities between young and deformed *N. furzeri*. In particular, the young and deformed killifish showed a spongy-like tissue, containing vacuolated notochord cells, which can be compared with the notochord. It is known that the notochord of teleost fish consists of two different cell types: epidermal-like cells in the outer layer, and an inner vacuolated cell core. The inner layer plays an especially important role in teleost, as the cells contain large lysosomal-derived vacuoles that can withstand high hydrostatic pressure. This feature is crucial for locomotion and survival in teleost fish. The development of the vertebral column in teleost fish is associated with a reorganization of the notochord, leading to the presence of non-vacuolated notochordal cells and extracellular lacunae, though these changes are not well-understood. Desmogleins, which are a conserved family of calcium-binding cadherin transmembrane proteins expressed in different tissues that undergo significant mechanical strain, are known to play a major role in maintaining tissue integrity. Seleit et al. described the presence of desmogleins in the notochord of medaka, the loss of which is associated with vacuolated cell defects and structural deformities in the notochord. This, afterwards, can lead to spinal malformations, as notochord abnormalities are associated with vertebral defects. Furthermore, it is described that the notochord, in particular the notochordal cells, plays a crucial part in vertebrae development and intervertebral disc formation, as their disappearance is linked to reduced repair capacity and intervertebral disc degeneration [26,27,28,29,30]. We expected to see similarities between old and deformed *N. furzeri*, as in human degenerative spine diseases and mammalian aging, a degradation of the intervertebral discs can be observed. Furthermore, as the deformed fish are closer in age to the old fish, we assumed that we would see similar results in the intervertebral area, which was not the case. We hypothesize that, with aging, *N. furzeri* develop a sarcopenic phenotype, which alters the mechanical loading of the vertebrae and might result in fibrosis of the intervertebral discs. This process seems to be missing in the deformed killifish. Irie et al. described that, in wavy medaka, the vacuolated notochord cells in the spongy-like tissue do not regress and, due to this, remain through the whole vertebral cavity and do not form the intervertebral disc [31]. Regarding the composition of vertebral bodies and intervertebral discs, we detected mainly type III collagen in the intervertebral area and trabecular structures of *N. furzeri*. As our polarization microscope lacks an integrated camera, we were not able to illustrate these findings, which is a limitation of our study. As described in other fish species, the bone matrix of the vertebral bodies consisted of type I collagen [32,33].

Similar findings were described by Irie et al. who compared healthy and wavy medaka (*Oryzia latipes*). The vertebral joints of wavy medaka showed spongy tissue containing vacuolated notochord cells but no intervertebral disc formation. Furthermore, they showed vertebral cavities filled with fibrous tissue and irregular-sized muscle fibers with a random alignment. In comparison, healthy medaka presented fibrous tissue in the central region and spongy tissue in the peripheral region, which consisted of regressing vacuolated notochord cells. The authors hypothesized that the spinal curvature might be due to genetic notochordal abnormalities [31].

Kranenbarg et al. investigated the effects of lordosis in sea bass (*Dicentrachus labrax* L.), comparing healthy and diseased fish. As lordosis is associated with an increased bone formation, diseased fish showed a high amount of chondroid bone. Furthermore, diseased sea bass showed densely fibrous chondroid tissue, which was not found in the healthy fish [34]. Comparing these findings with our investigations, *N. furzeri* did not show a high amount of chondroid bone.

Witten and colleagues described differences between healthy and deformed (short-tailed) Atlantic salmon (*Salmo salar*). Healthy salmon contained notochordal tissue in their intervertebral space, whereas deformed salmon showed cartilaginous connective tissue [35]. These findings are not comparable with the *N. furzeri*, as deformed killifish showed a spongy-like structure containing vacuolated notochord cells and no formation of fibrous tissue.

Grimmett et al. investigated sterile triploid grass carp (*Ctenophayngodon idella* (Valenciennes)) collected over a long period of time, which revealed spontaneous spinal deformities including kyphosis, scoliosis or rotation. The fish showed disorganized primarily mature mesenchymal tissues and asymmetrical spinal malformation [36]. In comparison to this, vertebral deformations in *N. furzeri* in our investigated population also occurred spontaneously.

Hayes et al. investigated the degenerative changes in the bone and cartilage of aged zebrafish (*Danio rerio*) and described an increased incidence of spinal deformities with age. Comparing younger and older zebrafish, 1- and 2-year-old fish showed vacuolated tissue of notochordal origin in the intervertebral area, which was not present in the 3-year-old zebrafish. Furthermore, they described the presence of osteophytes in the vertebral bodies of 2-year-old fish [37]. We detected similar findings in the *N. furzeri*, as older fish had only spongy-like tissue in the peripheral region containing regressing vacuolated notochord cells, but we could not see osteophytes in the vertebral bodies.

Comparing the phylogenetic background of the aforementioned fish, medaka and killifish are very closely related to each other, which would explain the similar histopathologic findings. The Atlantic salmon, which is not phylogenetically closely related to the killifish, showed differences in the intervertebral area. The grass carp, which is closely related with the zebrafish, also revealed spontaneously occurring vertebral deformations. The zebrafish, which is phylogenetically further related to the *N. furzeri*, also shows similar findings (i.e., missing vacuolated tissue of notochordal origin) in aging fish. Due to this, a connection between phylogeny and intervertebral disc characteristics could not be established.

The intervertebral discs of mice degenerate with age. Between 12 and 14 months, the intervertebral discs showed nucleus shrinkage and the focal loss of the lamellar structure present in the annulus [38]. Two-year-old mice showed a hypocellularity in their lumbar intervertebral discs. In general, the caudal lumbar intervertebral discs showed the most changes in aged mice [39]. Aging dogs showed changes in histology and decreasing proteoglycan content in the intervertebral discs [40]. Similarities could be detected in aging rabbits and sheep, including altered proteoglycan synthesis [41]. Furthermore, aging pigs displayed alterations in the biochemical properties of the lumbar discs [42]. In comparison to humans, notochordal cells present in the nucleus pulposus in some mammals, including mice or rats, do not vanish during their adult life [43]. Looking at bigger animals, like cows and sheep, similarities with humans can be detected, as the amount of notochordal cells decreases rapidly after birth [44]. In animal models, in comparison to humans, notochordal cells are larger, with vacuolated cells that are able to secrete hyaluronan [45].

Human intervertebral discs are crucial for providing flexibility to the spine and the transmission of mechanical load. In total, there are 25 intervertebral discs that are considered as the largest avascular structure of the human body. They are known to consist of an outer anulus fibrosus, which consists of dense fibrous connective tissue primarily of collagen type I. The anulus fibrosus surrounds a central nucleus pulposus, which is a gel-like liquid containing proteoglycan and water held together by a network of fine collagen type II and elastin fibers. In general, the intervertebral discs are connected by collagen fibers to the vertebral bodies and are able to balance the slow matrix turnover of synthesis and degradation [46,47,48,49]. The cells present in the anulus are elongated, parallel to the collagen fibers, and synthesize mostly collagen type I. Cells present in the nucleus are initially notochordal, but are replaced during childhood with rounded cells, which synthesize proteoglycans and type II collagen fibrils. The nucleus pulposus consists mostly of water, collagen type II and proteoglycans [50]. Intervertebral discs are described as undergoing age-related changes at the end of the first decade of life. These include the decline and/or proliferation of the nucleus of the pulposus cells, mild cleft formation, an increased number and extent of clefts and tears, cell density alterations, the presence of granular material, and matrix degeneration [51]. Furthermore, more than 50% of cells present in the discs are necrotic or show an apoptotic appearance [52]. During aging, the border between the annulus and the nucleus becomes less evident, and the nucleus pulposus changes from gel-like to more fibrotic [50]. The overall proteoglycan and water content decreases, mostly in the nucleus [53]. Moreover, intervertebral discs become more disorganized, leading to changes in their morphology [54]. Similarities were observed during our previous investigations into the *N. furzeri*, which showed a decrease of cartilage proteoglycans, in relation to the organic matrix, in female old *N. furzeri* [23]. This finding is also is in agreement with the potential development of human osteoarthritis, as cartilage proteoglycan content decreases [55,56]. Additionally, aging male *N. furzeri* showed a significantly lower bone density compared to younger killifish. Comparing the bone density of healthy and deformed fish, deformed *N. furzeri* had less dense vertebral bodies [23].

Degenerative disc diseases are associated with changes at the cellular level, including proliferation, cell cluster formation or increased cell death [57]. The reduction of large vacuolated notochordal cells in the nucleus pulposus is described as the initial step of disc degeneration [58]. Furthermore, reduced glycosaminoglycans and an increased cytokine and matrix metalloproteinases production occurs [59,60,61]. In scoliotic patients an ectopic calcification of the cartilage end plate can be observed, which affects the flow of nutrients and metabolites and can be associated with the low number of viable cells in scoliotic discs. In general, the intervertebral discs become deformed and narrowed due to an altered biomechanical environment [62]. Moreover, changes in the collagen turnover are described, as a higher amount of type I, III, VI, IX and X collagen and remodeling of collagen bundles is present. Intervertebral discs of scoliotic patients show sparse and disorganized elastic fiber networks [60,63,64]. Furthermore, degenerated intervertebral discs display a reduction in height and an abnormal mechanical response to loads [65]. The intervertebral area of deformed *N. furzeri* consists mostly of type III collagen, and type I collagen is present in the intervertebral bodies. 

As mentioned before, currently there is no cure for degenerative vertebral diseases, but rather treatment of the symptoms, like neck or low back pain or immobility, with pain medication and lifestyle amendments. To further understand the therapeutic efficacy of the treatment for these diseases, animal models are inevitable [3,49]. The advantage of fish models, especially for the *N. furzeri*, is their extremely short life cycle of three to seven months [21], which allows researchers to obtain results much faster than with rodent models. Furthermore, *N. furzeri* is an established model for accelerated aging, which shows different hallmarks of aging that are similar to those seen in mammals. As mentioned before, one of these is the presence of spinal curvature and reduction of locomotor activity [21,66,67]. Furthermore, previous publications showed that *N. furzeri* can be used also for interventional experiments, like, for example, dietary restriction [68] or acute confinement [69]. Looking at other fish models, medaka were used in several pharmacological interventions [70,71,72,73], as well as zebrafish [74,75,76]. These publications show that pharmacological intervention studies are also possible with fish models, which would serve as a basis for the investigation of therapeutic efficacies in *N. furzeri*. Especially for degenerative spine diseases, it is very important to further understand their pathophysiology and develop therapeutic strategies, as currently only symptomatic therapy approaches exist. As millions of people worldwide are affected by these diseases, the implementation of new animal models, like the *N. furzeri*, would be very beneficial.

## 5. Conclusions

We investigated intervertebral properties of the *N. furzeri*, which have never been documented before. Our study revealed age- and disease-related alterations of the vertebral discs in *N. furzeri*. When comparing these findings with other teleost fish, like medaka or zebrafish, similarities could be observed. Differences were also described in other fish models, like Atlantic salmon or sea bass. In previous investigations we saw a decrease of cartilage proteoglycan content in aging fish, which is similar to aging humans and a potential cause for the development of osteoarthritis. Further studies should investigate the utility of *N. furzeri* as a model for degenerative spine diseases.

## Data Availability

The data that support the findings of this study are available on request by the corresponding author.
